# Wake Up America: National Survey of Patients’ and Physicians’ Views and Attitudes on Insomnia Care

**DOI:** 10.3390/jcm12072498

**Published:** 2023-03-25

**Authors:** Ruth M. Benca, Suzanne M. Bertisch, Ajay Ahuja, Robin Mandelbaum, Andrew D. Krystal

**Affiliations:** 1 Department of Psychiatry and Behavioral Medicine, Wake Forest School of Medicine, Winston-Salem, NC 27103, USA; 2 Department of Medicine, Brigham and Women’s Hospital, Harvard Medical School, Boston, MA 02115, USA; 3 Idorsia Pharmaceuticals US Inc., Radnor, PA 19087, USA; 4 Department of Psychiatry and Behavioral Therapy, Weill Institute for Neurosciences, University of California San Francisco, San Francisco, CA 94107, USA; 5 Department of Neurology, Weill Institute for Neurosciences, University of California San Francisco, San Francisco, CA 94107, USA

**Keywords:** insomnia, treatment satisfaction, survey, primary care

## Abstract

While both patients and physicians consider sleep to be important, sleep health may not receive appropriate consideration during patient visits with health care professionals (HCPs). We completed the first large-scale survey of people with trouble sleeping (PWTS) and physicians who treat insomnia to understand their perspectives and potential discrepancies between them. The Harris Poll conducted online surveys of adult PWTS and HCPs (primary care physicians [PCPs] and psychiatrists) in the United States from September to October 2021. Respondents included 1001 PWTS, 300 PCPs, and 152 psychiatrists. Most HCPs agreed that sleep is critical to good health, yet very few reported routinely conducting full sleep histories on their patients. Approximately 30% of PWTS reported that their PCP never asks about sleep; zero HCPs in this survey reported “never” inquiring. Few HCPs reported being “very satisfied” with current treatment options; 50% of PCPs reported their patients being satisfied. Two-thirds of PWTS did not believe current treatment options adequately improved their sleep. This survey provides evidence that both PWTS and physicians agreed on the importance of sleep, but that treatment is often perceived as ineffective. This survey identifies a need for HCPs to address insomnia management and treatment gaps.

## 1. Introduction

Chronic insomnia disorder, defined as persistent difficulty initiating or maintaining sleep associated with daytime dysfunction despite adequate opportunity to sleep, affects approximately 10% of adults in the United States [1,2,3,4]. Further, 30–50% of adults report insomnia symptoms [1,2,3,4]. Insomnia is associated with a higher risk of developing psychiatric and medical conditions, such as anxiety, depression, hypertension, cardiovascular disease, and Alzheimer’s disease [4,5,6]. In addition to poor health outcomes, insomnia contributes to increased health care costs and utilization and lower worker productivity; more than 90% of insomnia-related costs, totaling an estimated $67 billion per year, are due to work absences and reduced productivity [2,7].

Despite the impact of insomnia on everyday life, this condition is often inadequately addressed and frequently undertreated [8,9]. As many as 40–50% of patients surveyed reported significant sleep disturbances to their primary care physicians (PCPs) [4,10]. However, PCPs, who provide the majority of sleep health care, may face constraints in managing insomnia given limited time, competing demands, and insufficient education on sleep disorders during their medical training [8,9,10,11]. As a result, persons with insomnia rarely receive interventions to address their symptoms. Additionally, the limited number of sleep specialists makes it difficult for PCPs to refer to a specialist. As a result, people with trouble sleeping (PWTS) often cannot receive care from a sleep expert [8,9,10,11].

Despite the high burden of insomnia, to date, there have been no extensive, quantitative studies examining both patients and health care professionals (HCPs) perspectives on insomnia and insomnia care [12], as prior surveys have typically only focused on perceptions, attitudes, and behaviors around sleep by the general population [13].

In this context, we conducted the Wake Up America: The Night & Day Impact of Insomnia (WUA) survey. WUA was the first national survey evaluating the experience of insomnia and approaches to its management to assess the perspectives of PWTS and HCPs who treat insomnia. This quantitative study also sought to further characterize themes from prior studies, such as the burden of insomnia, current solutions, level of satisfaction with current treatment options and patient/physician communication perceptions of PWTS and HCPs.

## 2. Materials & Methods

### 2.1. Study Design

WUA was conducted by The Harris Poll (Chicago, IL, USA; a research company; https://theharrispoll.com/ accessed on 12 February 2023) in the United States. The PWTS survey was conducted online from 27 September to 15 October 2021, and the physician survey was conducted online from 17 September to 14 October 2021. 

### 2.2. Survey Sample

All survey participants were recruited from multiple established online panels of consumers and physicians who had previously agreed to participate in market research. Potential participants received an email with a broad description of the study, inviting them to access the online survey via The Harris Poll. For the PWTS survey, the sample was targeted to a balanced mix of United States adults (based on United States Census data on sex, race/ethnicity, education, region, household income, size of household, and marital status) and included (i) those who reported that they had been diagnosed with insomnia by an HCP, or (ii) those who were likely to have insomnia based on their self-reporting of difficulty sleeping for ≥3 nights per week for at least 3 months. 

HCPs were targeted by their primary medical specialty and then further screened to verify whether they were actively licensed to practice in their state and whether their practice was in the United States. Eligible HCPs included PCPs specializing in family medicine, general practice or internal medicine, and psychiatrists.

### 2.3. Survey Content

The PWTS survey included 51 questions pertaining to sleep difficulties, interactions with HCPs, and treatment options. The physician survey included 34 questions pertaining to their approaches to and beliefs regarding insomnia diagnosis and management. All questions for both surveys were created through discussions with sleep experts around the condition of insomnia and the unmet need and by researching past surveys and polls about sleep or insomnia to identify gaps. The questions were then formulated and refined in collaboration with the poll experts at Harris Poll.

The full data set from these surveys can be accessed at https://www.wakeupamericasurvey.com/. Here we present the notable findings from the survey that focused on the burden of insomnia, current solutions, level of satisfaction with such treatment options, and patient/physician communications on these topics, which revealed potential differences in PWTS and HCP perceptions.

### 2.4. Statistical Analyses

The raw data were weighted by standard demographics, propensity weighting, and drop weighting to achieve a nationally representative sample of each respective group using methods proprietary to the Harris Poll. The data were also weighted to reflect the composition of the adult population aged ≥18 years and the population of the PCPs and psychiatrists, respectively. PWTS data for age by sex, race/ethnicity, education, region, household income, size of household, and marital status were weighted where necessary to align with their actual proportions in the population. Propensity score weighting was used to adjust for respondents’ propensity to be online. HCP data were weighted to reflect national estimates for years in practice, by gender, and by the region where necessary. Because this sample is based on those who agreed to participate in online surveys, no estimates of a theoretical sampling error could be calculated.

### 2.5. Ethical Considerations

The surveys were conducted under the standards and compliant practices of The Harris Poll. The questions did not include obtaining protected health information, and institutional review board approval was not required; however, PWTS and physicians provided informed consent to complete the surveys.

## 3. Results

### 3.1. Respondent Characteristics

PWTS respondents (*n* = 1001) had a mean age of 44.6 (SD 16.7) years; 54% were female; 58% were White, 20% were Hispanic, 12% were Black, and 6% were Asian. Of the PWTS, 18% reported being formally diagnosed with insomnia by their HCP, and 63% were employed (Table 1). For the HCP survey, 452 practitioners responded to the questionnaire; 66% (*n* = 300) were PCPs, and 34% (*n* = 152) were psychiatrists. PCPs had a mean age of 50.9 years and 59% were male. Psychiatrists had a mean age of 54.0 years, and 61% were male. PCPs and psychiatrists saw an average of 103.5 and 72.4 patients in a typical week (Table 1). 

### 3.2. Sleep as a Pillar of Health

For both survey groups, the vast majority of respondents agreed that, along with diet and exercise, sleep should be one of the pillars of health, as reported by 91% of PWTS and 98% of HCPs (PCPs 98%, psychiatrists 98%). In addition, PWTS reported that after a good night’s sleep, they were in a better mood (64%), were more productive (56%), and were better able to function during the day (50%). 

### 3.3. The Burden of Insomnia

**Figure 1:**
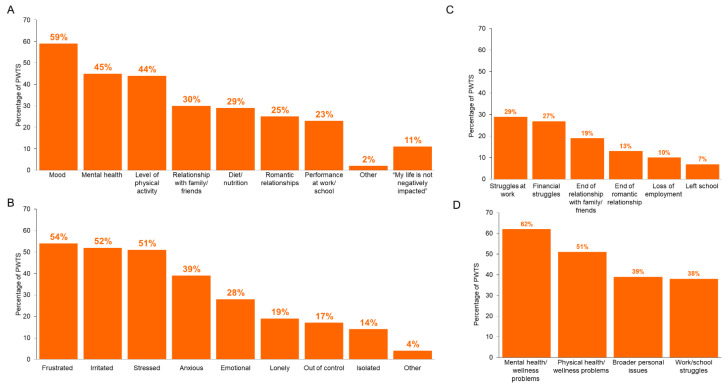
PPWTS reported consequences due to trouble sleeping/insomnia. PWTS reported (**A**) aspects of life negatively impacted by trouble sleeping/insomnia, (**B**) emotions due to trouble sleeping/insomnia, and (**C**) experiences due to trouble sleeping/insomnia. (**D**) A proportion of PWTS believes their life challenges are due to their trouble sleeping/insomnia. PWTS, people with trouble sleeping.

PWTS reported that the top three aspects of life negatively impacted by trouble sleeping/insomnia were mood, mental health, and physical activity level (Figure 1A). More than half of PWTS reported feeling frustrated (54%), irritated (52%) or stressed (51%) when they have trouble sleeping (Figure 1B). Over 30% of PWTS reported canceling plans at the last minute, getting into arguments with significant others, family members, or friends; forgetting appointments or daily activities; and reacting inappropriately to situations due to their trouble sleep difficulties.

Many PWTS experienced struggles at work, financial issues, or the ends of relationships, which they attribute to their trouble sleeping (Figure 1C). For example, of the PWTS who were employed (*n* = 580), 20% reported that they had lost 9+ hours/week at work due to trouble sleeping/insomnia. In addition, while a majority of PWTS believed that their mental health/wellness problems and physical health/wellness problems were due to their sleep problems, less than 40% of PWTS attributed their broader personal issues or work/school struggles to their sleeping difficulties (Figure 1D). 

Analogously, most PCPs (88%) and psychiatrists (85%) strongly agreed or somewhat agreed that people with sleeping difficulties do not realize their sleep issues’ toll on other aspects of their lives. While 60% of HCPs reported that their patients attribute their work or school struggles to their insomnia “always (or almost always)” or “often”, less than half of the HCPs reported that their patients were able to attribute their physical health or wellness problems or their broader personal issues to their insomnia.

### 3.4. Current Solutions for Insomnia

#### 3.4.1. Lifestyle

The majority of PWTS (86%) had made at least one lifestyle change, including minimizing caffeine intake in the late afternoon/evening (42%), exercising regularly (34%), or maintaining similar bedtimes and wake times every day (33%). Three-fourths of PWTS had purchased a lifestyle product, such as blackout curtains or a white noise machine, to improve their sleep, and these individuals spent an average of $285 in the past year on such items. 

While most PCPs (93%) and psychiatrists (89%) strongly or somewhat agreed with the statement “improved sleep hygiene is often the best solution to trouble sleeping/insomnia,” they also agreed that it is difficult for their patients to adhere to nonpharmacologic treatments (83% of PCPs, 86% of psychiatrists). Still, a large proportion of PCPs (52%) and psychiatrists (45%) reported recommending prescription sleep medication “only after exhausting other approaches (e.g., lifestyle changes, natural sleep aids, over-the-counter sleep aids)”.

#### 3.4.2. Nonprescription Treatments

**Figure 2:**
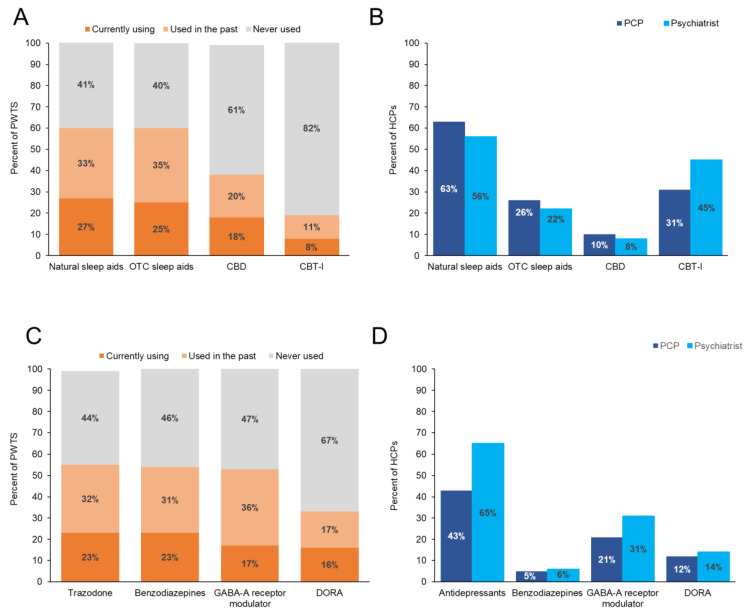
Use of prescription sleep medication and nonprescription treatment reported by PWTS and how often they are recommended by HCPs. (**A**) Use of natural sleep aids, OTC sleep aids, CBD, and CBT-I in PWTS who have ever used a prescription sleep medication and (**B**) percentage of PCPs/psychiatrists who always (or always)/often recommend nonprescription treatments. (**C**) Use of trazodone, benzodiazepines, GABA-A receptor modulators, and DORAs in PWTS who have ever used a prescription sleep medication and (**D**) the percentage of PCPs/psychiatrists who always (or always)/often prescribe treatments. Due to rounding completed by the Harris Poll, percentages may add up to more than 100%. CBD, cannabidiol; CBT-I, cognitive behavioral therapy for insomnia; DORA, dual orexin receptor antagonist; GABA-A, γ-aminobutyric acid type A; HCP, health care professional; OTC, over the counter; PCP, primary care physician; PWTS, people with trouble sleeping.

Items, such as natural remedies and over the counter (OTC) medications, were each reported to be used at some point by 60% of PWTS. Notably, cannabidiol (CBD) was reportedly used by 39% of PWTS. However, the only nonmedication treatment for sleep recommended by the American Academy of Sleep Medicine (AASM) [14,15] is cognitive behavioral therapy for insomnia (CBT-I), which was reported as being used by only 19% of PWTS (Figure 2A). 

On the provider side, the most common nonprescription treatments that HCPs “always (or almost always)” or “often” recommend to patients were reported to be natural sleep aids (e.g., melatonin, valerian root) (63% of PCPs, 56% of psychiatrists) and CBT-I (31% of PCPs, 45% of psychiatrists). Furthermore, roughly one-fourth of HCPs (26% of PCPs, 22% of psychiatrists) recommended OTC sleep aids, and 10% of PCPs and 8% of psychiatrists recommended CBD “always (or almost always)” or “often” to their patients with trouble sleeping/insomnia (Figure 2B). 

#### 3.4.3. Prescription Sleep Medications

Regarding the consumption of prescription sleep medications, while over 50% of PWTS believed that there is a stigma associated with taking such medicines, just 29% of those diagnosed with insomnia or who currently see a PCP (*n* = 922) reported taking a prescription drug. Among the medications currently being taken by PWTS were: (1) trazodone (23%), (2) benzodiazepines (e.g., alprazolam [Xanax], diazepam [Valium], estazolam [Prosom]) (23%), (3) nonbenzodiazepine γ-aminobutyric acid type A (GABA-A) receptor positive allosteric modulators (e.g., zolpidem [Ambien], eszopiclone [Lunesta]) (17%), and (4) medications in the dual orexin receptor antagonist (DORA) class (e.g., suvorexant [Belsomra], lemborexant [Dayvigo]) (16%). The most common prescription sleep medications used either in the past or currently by PWTS were: (1) trazodone (56%) and (2) benzodiazepines (54%) (Figure 2C). 

Following a comparable pattern, when HCPs prescribe sleep medicines for insomnia, 43% of PCPs and 65% of psychiatrists reported “always (or almost always)” or “often” treating with antidepressants (e.g., trazodone, fluoxetine [Prozac]), followed by GABA-A receptor modulators (e.g., Ambien), DORAs (e.g., Belsomra), and benzodiazepines (e.g., temazepam) (Figure 2D). Notably, almost all PCPs (92%) and psychiatrists (99%) had at some point prescribed trazodone off-label to patients to specifically treat trouble sleeping/insomnia.

### 3.5. Level of Satisfaction with Current Treatment Options

**Figure 3:**
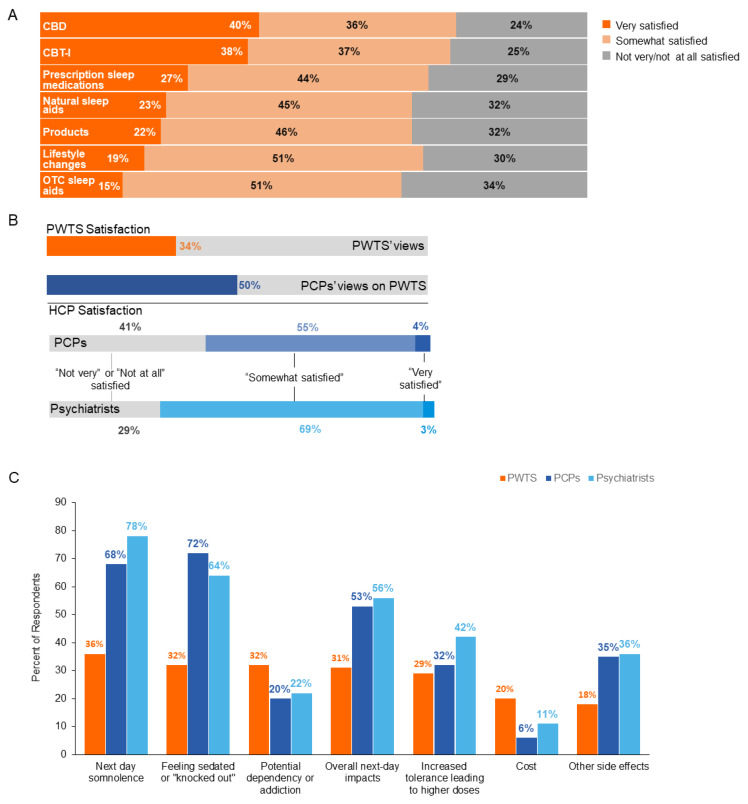
PWTS satisfaction with each treatment/solution currently being used, overall satisfaction with current pharmacologic treatment options, and the top concerns about OTC sleep aids for trouble sleeping/insomnia for PWTS, PCPs, and psychiatrists. (**A**) PWTS reported satisfaction with their current treatment. (**B**) PWTS overall satisfaction with current pharmacologic treatments, PCPs’ views on PWTS satisfaction, and HCP overall satisfaction. (**C**) Top concerns of PWTS, PCPs, and psychiatrists about OTC sleep aids for trouble sleeping/insomnia. CBD, cannabidiol; HCP, health care professional; OTC, over the counter; PCP, primary care physician; PWTS, people with trouble sleeping.

Among the various nonprescription treatments for insomnia, CBD and CBT-I had the highest proportion of PWTS (40% and 38%, respectively) reporting being “very satisfied” with their treatments, while with lifestyle changes or OTC sleep aids, less than 20% of PWTS were “very satisfied” (Figure 3A). 

Almost all HCPs (95% of PCPs, 92% of psychiatrists) strongly agreed or somewhat agreed that they wish they could do more to help treat their patients with trouble sleeping/insomnia. In addition, most PCPs (97%) and psychiatrists (93%) would like to learn more about ways to help their patients with trouble sleeping/insomnia, and they agreed that more medical education for HCPs on insomnia would be valuable (97% of both PCPs and psychiatrists). 

Furthermore, a notable majority (66%) of PWTS surveyed agreed with the statement, “I don’t think current treatment options can help improve my insomnia/trouble sleeping,” but PCPs and psychiatrists reported that they believe that approximately half (average 49.0% and 54.4% of patients, respectively) of their patients are indeed satisfied with their current treatments. And most HCPs (59% of PCPs, 71% of psychiatrists) reported that they are “satisfied” with the current treatment options available for their patients, though very few (4% of PCPs, 3% of psychiatrists) reported being “very satisfied” (Figure 3B).

#### 3.5.1. Nonprescription Treatments

Regarding OTC sleep aids for trouble sleeping/insomnia, such as antihistamines, Benadryl, or ZzzQuil, the top 5 concerns of PWTS were: (1) next-day somnolence (feeling sleepy and lethargic in the morning), (2) “feeling sedated or knocked out,” (3) potential dependency or addiction, (4) overall next-day impacts, and (5) tolerance leading to higher doses (Figure 3C). HCPs largely agreed with the reports of PWTS in that their top two concerns for their patients were also next-day somnolence and “feeling sedated or knocked out”, but HCPs reported neither potential dependency nor addiction in their top 5 concerns; rather, their concerns included other side effects (e.g., sleepwalking, nightmares) (Figure 3C).

#### 3.5.2. Prescription Sleeping Pills

In contrast, common concerns about prescription sleep medications by both PWTS and HCPs were: (1) potential dependency or addiction; (2) next-day somnolence; (3) tolerance leading to higher doses; (4) “feeling sedated or knocked out”; and (5) overall next-day impacts.

### 3.6. Patient/Physician Communication about Sleeping Problems and Insomnia

More than half (57%) of PWTS who were not formally diagnosed with insomnia (*n* = 834) stated that they have not spoken to their HCP about their trouble sleeping. Their rationale for not discussing their troubles sleeping with their HCP included that they “don’t want to be prescribed sleep medications” (31%), “never thought about bringing it up” (26%), “don’t think it’s a big deal” (24%), or “should be able to fix my sleep troubles on my own” (24%).

**Figure 4:**
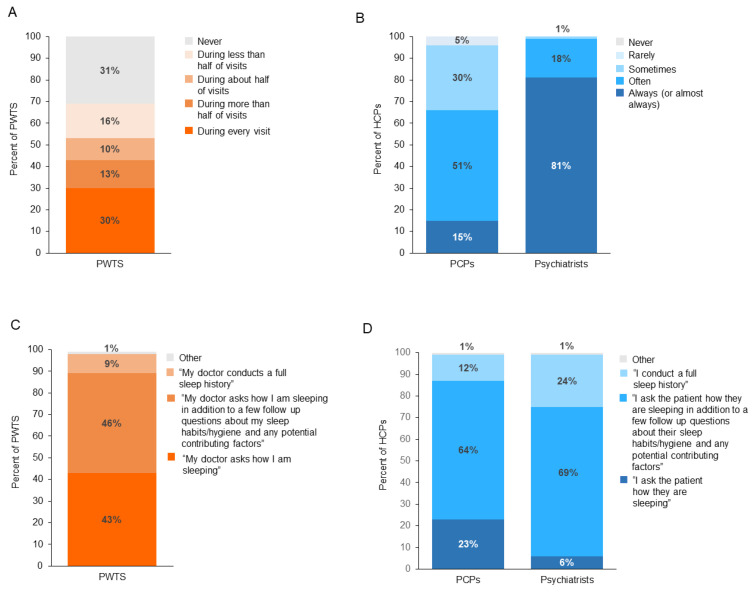
Perceived differences between PWTS and HCPs on sleep discussions. (**A**) PWTS reported the frequency of their PCP asking about sleep and sleep habits during routine visits (**B**), and the frequency HCPs reported asking their patients about sleep and sleep habits during routine visits. (**C**) The types of sleep conversations PWTS report having with PCPs (**D**) and the types of conversations HCPs report having with their patients during routine visits. Due to rounding completed by the Harris Poll, percentages may add up to more than 100%. HCP, health care professional; PCP, primary care physician; PWTS, people with trouble sleeping.

Among the PWTS who see a PCP (*n* = 901), 43% reported that their PCP queries them about their sleep and sleep habits “during every visit” or “during more than half of visits” (Figure 4A), while from the physician perspective, 66% of PCPs and 99% of psychiatrists, respectively, claim that they “always (or almost always)” or “often” do so during routine visits (Figure 4B). In contrast, about 30% of PWTS reported that their PCP never asks about sleep, yet no PCPs or psychiatrists in this survey admitted to “never” asking such questions. Nonetheless, among the PWTS who do discuss sleep during routine visits (*n* = 667), the most common aspects of insomnia discussed were the impacts on mental health (53%), physical health (48%), and daytime functioning (44%). 

Additionally, PWTS reported that even when their PCP did ask about sleep, a full sleep history (defined as asking about sleep patterns, daytime sleepiness, sleep quality, and sleep history over time) was rarely (9%) conducted (Figure 4C); but when physicians were asked about how they conduct discussions with their patients about sleep, 12% of PCPs claimed that they do conduct full sleep histories during routine visits (as did 24% of psychiatrists) (Figure 4D).

## 4. Discussion

According to the American Heart Association, this first large-scale survey of PWTS and HCPs provided evidence that both groups agreed on the importance of sleep, an essential component of cardiovascular health [16]. In addition, the majority of PWTS, PCPs, and psychiatrists agreed with the characterization that sleep should be considered a key pillar of health. However, in practice, even while exercise, diet, and sleep have all been shown to be important for both physical and mental health [16,17,18,19], sleep has still not become a priority for most HCPs [8,11,20].

Approximately 25 million adults in the United States have insomnia [1,3,4]. Consistent with other studies, PWTS in this survey reported multiple aspects of their life being negatively impacted by their trouble sleeping/insomnia [12,21]. Other studies have found that patients describe daily life as an effort or struggle and that insomnia prevents them from being their “desired self” [4,21]. This survey’s results reflect that over one-fourth of PWTS struggled at work or financially, and 10% lost their employment due to their trouble with sleeping/insomnia, consistent with prior studies of patients with insomnia [2,21]. 

The results of WUA indicate that many PWTS are often making lifestyle changes to improve their sleep hygiene, such as minimizing caffeine intake, exercising regularly, and maintaining similar bedtimes and wake times [15,18]. However, as could be expected, less than 20% of PWTS are “very satisfied” with such lifestyle changes as a solution for their insomnia. Further, most HCPs surveyed stated that they viewed improved sleep hygiene as the best solution to improving sleep among their patients with insomnia, despite the AASM’s recommendations stating that clinicians should not use sleep hygiene as a single-component therapy for the treatment of chronic insomnia [15]. This indicates a notable clinical translational gap between guideline recommendations and the delay in “front-line” medical caregivers providing patients with evidence-based care. While this survey did not provide data to explain this, this gap may be due to a lower emphasis on insomnia in medical education curricula compared to other disorders with better-followed guidelines. Of note, many nonspecialists in sleep medicine confuse sleep hygiene with CBT-I, and because the terms were not specifically defined in the WUA survey, these results may represent varying opinions regarding HCP education about sleep hygiene. 

PWTS still report that they are desperately seeking treatments for their sleeping difficulties, even though the majority claim they do not believe that current treatment options would help. Moreover, a small but notable proportion of HCPs agreed that current treatment options are suboptimal, and most may have more risks than benefits.

According to current guidelines, CBT-I is the standard and recommended first-line therapy for patients with chronic insomnia [15,22]. Access to CBT-I is also limited due to a lack of therapists, stigmatization, high costs, and lack of awareness [20,23,24]. However, we found that a relatively high proportion of HCP respondents in this survey stated that they refer their patients for CBT-I. This contrasts with other studies reporting that referrals for CBT-I or psychological treatment are uncommon (5–10%) [8,20], which is consistent with our finding that 8% of PWTS reported currently receiving CBT-I therapy. Given the known access challenges, our findings may represent overestimation by the HCPs in WUA, misperception of the definition of CBT-I (mistaking sleep hygiene for CBT-I), representativeness of our surveyed practitioners (may live in regions with better CBT-I access), and/or the fact that a very high rate of referrals may not have translated to real-world access for patients, unbeknownst to the referring HCPs. Regardless, our study reinforces the need to broaden access to CBT-I. Future studies should examine the potential access challenges to CBT-I.

Although several nonprescription and prescription medicines might be used to treat insomnia, and while HCPs felt that half of their patients are satisfied with current treatment options, two-thirds of the PWTS surveyed did not believe that the current treatment options available could adequately improve their trouble sleeping. This view by PWTS contrasts with other published efficacy and safety data for a number of US Food and Drug Administration (FDA)-approved medications. It may reflect a bias on the part of PWTS regarding only those treatment options offered to them in the past. There were, however, high rates of alignment between PWTS and HCPs with respect to the trial of OTC sleep aids (lack of their effectiveness notwithstanding) and to the top concerns that some of these can lead to next-day somnolence and/or “feeling sedated or knocked out.” This aligns with the AASM guidance of not using OTC sleep aids because these medications have not been effective in managing insomnia and/or have significant potential for harm [14].

While there are a number of prescription medication options available for PWTS, each has its benefit-risk profile. Among the hypnotic medications, the most common are benzodiazepines, nonbenzodiazepines (also known as “Z-drugs”), DORAs, and the off-label use of trazodone to treat insomnia. 

Regarding these medicines, HCPs generally express reservations about the use of benzodiazepines due to their side effects and concerns regarding dependency [14]. Consistent with this view, relatively few PCPs and psychiatrists reported “often” or “always (or almost always)” prescribing this class. Nonetheless, about one-fourth of PWTS surveyed reported taking a benzodiazepine currently; further, one-third of PWTS claim to have used a benzodiazepine at some point. It should be noted that this value from WUA is higher than the 16% rate reported in other studies [8,25].

Compared to reported benzodiazepine use, fewer PWTS in WUA reported currently receiving a Z-drug, while about half of PWTS had previously used this class of medications at some point. From the prescribers’ point of view, less than one-third of HCPs “always (or almost always)” or “often” prescribe these medications per WUA. As a reference, the literature demonstrates a wide range of rates of use of these medications, indicating that 20–58% of patients with insomnia in the United States are prescribed nonbenzodiazepines [8,25,26,27]. 

Other options for patients with insomnia now include two newer medicines in the DORA class, lemborexant and daridorexant. These were approved by the FDA after the most recent updates to the AASM and American College of Physicians (ACP) guidelines in 2017 and 2016, respectively. Therefore, only suvorexant, the first member of the DORA class, was included in those most recent guidelines. DORAs might theoretically have the same abuse potential as nonbenzodiazepines and can be associated with somnolence, dizziness, or sleep paralysis [28,29]. Medications of the DORA class were reported in WUA to be used by one-third of PWTS, which is higher than the estimated <20% nationally [25]. 

A 2023 update by the Insomnia Working Group highlighted that challenges exist in treating insomnia with commonly used off-label drugs, including trazodone [30]. Per the Insomnia Working Group, trazodone is the most frequently prescribed medication for insomnia in the primary care setting in the United States [30]. The WUA surveys are consistent with this report, showing that this antidepressant was the top prescribed medication for insomnia among respondents; this is also consistent with other studies citing its use in patients with insomnia (18–55%) [25,26]. However, this use notably contradicts the recommendations of the AASM guidelines, which state that trazodone should not be used to treat insomnia, given limited efficacy data and potential risks such as serious cardiovascular side effects due to an overdose [14,31,32]. 

Nonmedication treatment options other than CBT-I are not included in the AASM guidelines [14,15]. The increasing availability of nonprescription CBD products may lead to more patients using these for trouble sleeping [30]. More than one-third of PWTS in this survey reported using CBD. In another survey of the general US population with a similar demographic background as WUA, approximately 26% of respondents used CBD products [33]. Evidence supports the potential use of medical marijuana and related products for conditions like pain or anxiety, which are commonly comorbid with insomnia [30].

We found that despite the increasing awareness of the importance of good sleep to good health, [16,17,18,19] over half of PWTS have not discussed their trouble sleeping with an HCP. Many PWTS reported that they “don’t think it’s a big deal” or “should be able to fix my sleep troubles on my own”, suggesting that insomnia is a hidden problem, despite having significant impacts on daily life [12,21]. This view was confirmed in the survey as insomnia medications were viewed by PWTS as having an associated stigma. This viewpoint is consistent with findings from other studies, which have similarly highlighted that patients usually felt misunderstood by others or had a sense of resignation and hopelessness about insomnia and its treatment [12,34]. Some research has suggested that patients might prefer nonpharmacological treatments with long-term benefits, while their clinicians, in contrast, may perceive that patients often prefer a “quick fix” and, as a result, readily prescribe pharmacotherapies. This disconnect could be related to the limited availability and accessibility of effective treatment options through primary care [34]. 

Still, it behooves physicians to ask their patients about sleep issues, recognize and diagnose insomnia, and offer the most appropriate treatment regimen. Yet it was revealed that of the PWTS who regularly see a PCP, one-third reported that their PCP, in fact, never asks about their sleep; when the latter question was posed to the physicians, no HCPs reported “never.” This is a key example of a disconnect in the patient/physician dialogue. This issue requires further study to elucidate how to improve such understanding and foster better patient/physician communication. 

Despite its size and scope, there were a number of limitations of this study. This survey was not necessarily based on an exact representative sampling of the general population. WUA was limited to persons who enlisted in the Harris poll, had online access, and were conversant in English. Accordingly, there might be sampling bias. In addition, the PWTS surveyed were not the patients of the HCPs surveyed. PWTS self-reported their sleep problems and insomnia diagnosis. Clinical types of insomnia were not determined, and future studies could determine whether the attitudes of the PWTS population differ across the various types of insomnia. In addition, future studies could examine the views on insomnia care of the general population compared to those with trouble sleeping. The WUA survey was also not designed to explore the demographic characteristics of respondents related to their answers to individual questions. Furthermore, the respondents were primarily middle-aged; different views on education on sleep and expectations from treatment could vary by age group for both HCPs and PWTS. Finally, the survey was conducted during the COVID-19 pandemic.

## 5. Conclusions

PWTS and HCPs agreed on the importance of sleep to health, yet relatively few physicians conduct a full sleep history, and less than half of patients discuss their sleep problems with their HCP. 

PWTS and HCPs agreed that there are limitations with the currently available treatment options, although a greater proportion of PWTS surveyed believed this. Furthermore, many PWTS report that they perceive a stigma attached to the use of insomnia medications. Therefore, more education is needed for HCPs on how to diagnose and treat insomnia and for PWTS to understand appropriate and effective treatment options. Future studies may be done to determine potential continuing medical education preferences among HCPs and PWTS and determine what sleep-related education HCPs have received. 

Overall, there were some key areas of alignment between the viewpoints of PWTS and HCPs. Still, there were also some very notable discrepancies, especially those related to patient/physician communication regarding both the recognition and diagnosis of insomnia and the choice of the most suitable treatment options. Further work is needed to identify the reasons for such discrepancies and examine the population of PWTS and HCPs who do not consider sleep a pillar of health. These are important as they indicate particular areas in which, likely, improved education and medical care can better meet the needs of patients regarding sleep, and a further understanding can improve the overall health and wellness of the overall population of the United States.

## Figures and Tables

**Table 1:** PWTS and HCP demographic characteristics.

PWTS Characteristics		
Age, mean (SD), years	44.6 (16.7)	
Gender, %		
Male	44	
Female	54	
Other ^a^	3	
Race, %		
White	58	
Hispanic	20	
Black or African American	12	
Asian ^b^	5	
Pacific Islander	1	
Native American or Alaskan Native	1	
More than one race	3	
US Region, %		
South	40	
West	24	
Northeast	18	
Midwest	18	
Conditions diagnosed by HCP, %		
Anxiety/anxiety disorder	32	
High blood pressure	30	
Depression	29	
High cholesterol	21	
Insomnia	18	
Chronic pain	17	
Diabetes	16	
Obesity	9	
Other psychiatric disorder	6	
None	24	
Employment status, %		
Employed	63	
Retired	16	
Student	3	
Stay-at-home spouse or partner	6	
Age first experienced trouble sleeping, mean, years	30.9	

**HCP Characteristics**	**PCPs**	**Psychiatrists**
Age, mean (SD), years	50.9 (11.9)	54.0 (13.1)
Gender, %		
Male	59	61
Female	36	38
Years in practice, %		
0 to 2 years	6	11
3 to 10 years	24	12
11 to 20 years	29	25
21 to 30 years	20	26
31+ years	21	26
Types of medical practice, %		
Mostly office- or clinic-based	87	66
Mostly hospital- or lab-based	3	9
Exclusively hospital- or lab-based	2	6
Mostly long-term care facility-based	1	3
Mostly hospice-based	1	1
Equally hospital- and office/clinic-based	7	15
Patient population ages, %		
≤18 years (pediatric)	-	3
19 to 64 years (adult)	11	38
≥19 years (adult and geriatric)	33	39
≥65 years (geriatric)	8	3
All ages	49	16
The number of patients seen in a typical week mean	103.5	72.4
The number of patients with trouble sleeping seen each month mean	58.5	94.6
The number of patients diagnosed with insomnia seen each month mean	45.6	77.2

^a^ Others included transgender (1%), nonbinary or gender nonconforming (1%) and those who preferred not to answer (1%). ^b^ Asian included Filipino (2%), Chinese (2%), and South Asian (1%). HCP, health care professional; PWTS, people with trouble sleeping; SD, standard deviation. Bold for the row “HCP Characteristics”: Above this section was strictly PWTS characteristics (which was bolded), here we are changing the table headers and are denoting it with bolded text and a thick line above. Thick bar line: this separates the PWTS characteristics from the HCP characteristics.

## Data Availability

The full data set from these surveys can be accessed at https://www.wakeupamericasurvey.com/.
